# Supplementary material for: Genome-Wide Identification of the Oxidative Stress 3 (OXS3) Gene Family and Analysis of Its Expression Pattern During Ovule Development and Under Abiotic Stress in Cotton
Source: Biology (Basel). 2024 Nov 6;13(11):903. doi: 10.3390/biology13110903 (PMC11591572; doi:10.3390/biology13110903)
Supplement: Supplementary file 1 [file biology-13-00903-s001.zip › Table S1 Specific primers for OXS3 gene family on qRT-PCR experiments.pdf]

Table S1 Specific primers for OXS3 gene family on qRT-PCR experiments

Gene ID	Primer sequences
GhOXS3-1F	TGTAAAAGGTATGGGGGTGGA
GhOXS3-1R	TAGATGAGATAGAGGGGGCCG
GhOXS3-3F	TGCAAAGCCTGAAGATCCCT
GhOXS3-3R	GAGGCTTTGGTCGAGCAGTT
GhOXS3-5F	GCCGCTCCTTGCTTTGAATG
GhOXS3-5R	TCAACTTGTACTTGCGGTTCA
GhOXS3-6F	TTTAGCAAGCGTGAGGAGCA
GhOXS3-6R	AACCTCGACTGGAACAACCC
GhOXS3-10F	GGAAGTGCAATGGCTTTCGG
GhOXS3-10R	GACAAGGAGAAAGACCGGCA
GhOXS3-12F	ACACCGTTGAAGAACTCGCA
GhOXS3-12R	CTCCCTAGGCATGACGATGA
GhOXS3-18F	CGCTCTAAGGATACGATTCCCTC
GhOXS3-18R	GCCTTTACTTTCCCCGGATG
GhOXS3-19F	CTGCATCATCATCGAATGGGC
GhOXS3-19R	ACACGAACCACTTTCTTTGGT
GhOXS3-21F	GCGGGTAATGAATTGCGCT
GhOXS3-21R	CCACCGTCCGATGATATTCCG
GhOXS3-22F	TGTGAACACCATTGAAGAACTTGC
GhOXS3-22R	CTTCCTCCCTGGACACGATG
GhUBQ7F	GAAGGCATTCCACCTGACCAAC
GhUBQ7R	CTTGACCTTCTTCTTCTTGTGCTTG
