# Supplementary material for: Genome-Wide Identification of the Oxidative Stress 3 (OXS3) Gene Family and Analysis of Its Expression Pattern During Ovule Development and Under Abiotic Stress in Cotton
Source: Biology (Basel). 2024 Nov 6;13(11):903. doi: 10.3390/biology13110903 (PMC11591572; doi:10.3390/biology13110903)
Supplement: Supplementary file 1 [file biology-13-00903-s001.zip › Table S2 The identification and physicochemical properties of OXS3 genes in cotton.pdf]

Table S2 The identification and physicochemical properties of *OXS3* genes in cotton

Gene Name	Gene ID	Genomics Positions	Physicochemical property				Group
			Length	MW/kD	pI	GRAVY	
GhOXS3-1	GH_A02G1392	A02:83647242-83647643	105	11.79	10.28	-0.627	1
GhOXS3-2	GH_A05G0337	A05:3303796-3304629	148	15.92	10.3	-0.499	3
GhOXS3-3	GH_A05G0339	A05:3310439-3311525	246	26.78	6.84	-0.838	3
GhOXS3-4	GH_A05G0386	A05:3654561-3655202	185	20.25	5.91	-0.646	3
GhOXS3-5	GH_A05G0649	A05:5864935-5865903	238	25.23	4.85	-0.48	3
GhOXS3-6	GH_A06G0735	A06:16153564-16154192	176	18.98	8.5	-0.576	1
GhOXS3-7	GH_A06G0876	A06:224300756-22431217	305	33.96	7.6	-0.529	3
GhOXS3-8	GH_A06G1340	A06:76118585-76119370	232	25.30	4.96	-0.759	3
GhOXS3-9	GH_A07G0097	A07:1052223-1053330	145	15.79	9	-0.611	1
GhOXS3-10	GH_A07G0154	A07:1613133-1615028	281	30.52	5.98	-0.473	3
GhOXS3-11	GH_A07G0197	A07:1986644-1987498	255	27.80	5.84	-0.575	3
GhOXS3-12	GH_A09G0996	A09:61155582-61156403	122	13.56	10.26	-0.461	1
GhOXS3-13	GH_D05G0344	D05:2832969-2834060	246	26.87	6	-0.853	3
GhOXS3-14	GH_D05G0389	D05:3139525-3140165	185	20.17	7.67	-0.598	3
GhOXS3-15	GH_D05G0646	D05:5138910-5139857	231	24.65	4.89	-0.545	3
GhOXS3-16	GH_D06G0712	D06:11883555-11884195	180	19.39	8.71	-0.593	1
GhOXS3-17	GH_D06G0860	D06:15233862-15234824	244	26.72	8.35	-0.827	3
GhOXS3-18	GH_D06G1175	D06:25646986-25647777	232	25.28	4.96	-0.744	3
GhOXS3-19	GH_D07G0103	D07:1018061-1018562	139	15.30	9.54	-0.539	1
GhOXS3-20	GH_D07G0163	D07:1588268-1590089	248	26.91	5.82	-0.648	3
GhOXS3-21	GH_D07G0209	D07:1963557-1964415	255	27.74	5.58	-0.569	3
GhOXS3-22	GH_D09G0948	D09:34141191-34142176	181	20.16	8.61	-0.648	1
GbOXS3-1	GB_A01G0043	A01:288897-289282	96	11.38	9.74	-1.286	2
GbOXS3-2	GB_A05G0342	A05:3544746-3545579	148	15.89	10.3	-0.515	3
GbOXS3-3	GB_A05G0344	A05:3548282-3549368	246	26.78	6.84	-0.838	3
GbOXS3-4	GB_A05G0391	A05:3891801-3892442	185	20.24	5.91	-0.658	3
GbOXS3-5	GB_A05G0652	A05:6152620-6153564	230	24.38	4.91	-0.474	3
GbOXS3-6	GB_A06G0769	A06:15771469-15772097	176	18.98	8.5	-0.576	1
GbOXS3-7	GB_A06G0907	A06:22029818-22030960	305	33.96	7.6	-0.529	3
GbOXS3-8	GB_A06G1380	A06:70775624-70777052	253	27.62	5	-0.703	3
GbOXS3-9	GB_A07G0087	A07:1095561-1096669	145	15.81	9.17	-0.62	1
GbOXS3-10	GB_A07G0147	A07:1736490-1738385	281	30.50	5.98	-0.469	3
GbOXS3-11	GB_A07G0191	A07:2131334-2132942	198	21.53	5.37	-0.544	3
GbOXS3-12	GB_A09G1108	A09:57009725-57011843	180	19.99	7.72	-0.528	1
GbOXS3-13	GB_D05G0342	D05:3075779-3076537	118	12.74	9.94	-0.153	3
GbOXS3-14	GB_D05G0344	D05:3079136-3080227	246	26.87	6	-0.853	3
GbOXS3-15	GB_D05G0391	D05:3400475-3401115	185	20.24	8.77	-0.617	3
GbOXS3-16	GB_D05G0646	D05:5333888-5334841	233	24.85	4.89	-0.527	3
GbOXS3-17	GB_D06G0752	D06:11451352-11451989	179	19.31	8.71	-0.592	1
GbOXS3-18	GB_D06G0905	D06:14819789-14820952	311	34.49	8.85	-0.418	3

Gene Name	Gene ID	Genomics Positions	Physicochemical property				Group
			Length	MW/kD	pI	GRAVY	
GbOXS3-19	GB_D06G1225	D06:25021374-25022756	257	28.10	5.1	-0.653	3
GbOXS3-20	GB_D07G0096	D07:1008359-1008884	147	16.09	9.36	-0.489	1
GbOXS3-21	GB_D07G0155	D07:1575486-1577303	248	26.95	6.74	-0.667	3
GbOXS3-22	GB_D07G0199	D07:1956147-1957845	198	21.45	5.2	-0.523	3
GbOXS3-23	GB_D09G0957	D09:35532391-35533405	190	21.10	7.67	-0.665	1
GaOXS3-1	Ga01G0045	Chr01:306923-307307	96	11.39	9.74	-1.283	2
GaOXS3-2	Ga02G0678	Chr02:26757700-26758359	169	18.95	9.03	-0.611	1
GaOXS3-3	Ga05G0358	Chr05:3275628-3276463	148	15.87	10.02	-0.405	3
GaOXS3-4	Ga05G0360	Chr05:3279186-3280262	243	26.39	8.53	-0.805	3
GaOXS3-5	Ga05G0677	Chr05:59621896-5963119	225	23.93	4.91	-0.49	3
GaOXS3-6	Ga06G0691	Chr06:11806910-11807544	178	19.15	8.5	-0.579	1
GaOXS3-7	Ga06G0883	Chr06:20538829-20539956	300	33.36	7	-0.466	3
GaOXS3-8	Ga06G1235	Chr06:48957825-48958611	232	25.28	4.96	-0.747	3
GaOXS3-9	Ga07G0101	Chr07:1169190-1169685	137	14.91	9.26	-0.488	1
GaOXS3-10	Ga07G0163	Chr07:1806801-1808640	248	26.69	5.48	-0.635	3
GaOXS3-11	Ga07G0207	Chr07:2232495-2233352	255	27.80	5.84	-0.575	3
GaOXS3-12	Ga09G0966	Chr09:61342882-61343917	190	21.18	8.69	-0.574	1
GrOXS3-1	Gorai.001G009600	Chr01:911417-912172	137	14.98	9.46	-0.558	1
GrOXS3-2	Gorai.001G015500	Chr01:1459720-1462084	248	26.98	5.99	-0.645	3
GrOXS3-3	Gorai.001G019700	Chr01:1834375-1835941	255	27.74	5.58	-0.569	3
GrOXS3-4	Gorai.002G005100	Chr02:322999-323381	96	11.39	9.68	-1.282	2
GrOXS3-5	Gorai.003G061300	Chr03:10926457-10927092	161	18.07	9.57	-0.606	1
GrOXS3-6	Gorai.006G096500	Chr06:33591891-33593672	190	21.10	7.67	-0.667	1
GrOXS3-7	Gorai.009G035600	Chr09:2651196-2652413	123	13.45	10.03	-0.059	3
GrOXS3-8	Gorai.009G040300	Chr09:2975008-2976312	185	20.14	7.66	-0.587	3
GrOXS3-9	Gorai.009G067000	Chr09:4791627-4793166	230	24.57	4.96	-0.518	3
GrOXS3-10	Gorai.010G078200	Chr10:11361428-11363048	177	19.08	8.71	-0.576	1
GrOXS3-11	Gorai.010G092500	Chr10:14849905-14851126	311	34.47	8.85	-0.44	3
GrOXS3-12	Gorai.010G122000	Chr10:25045906-25047407	232	25.33	5.06	-0.75	3
